# Body mass index and sex differences for mortality in hospitalized COVID-19 patients: a path analysis using a brazilian national database

**DOI:** 10.1186/s12889-023-16218-1

**Published:** 2023-08-29

**Authors:** Erika Cardoso dos Reis, Elma Lúcia de Freitas Monteiro, Joilson Meneguci, Phillipe Rodrigues, Alexandre Palma, Jair Sindra Virtuoso Junior, Sonia Regina Lambert Passos, Maria Angelica Borges dos Santos

**Affiliations:** 1 Escola de Nutrição, Departamento de Nutrição Clínica e Social, Universidade Federal de Ouro Preto, Minas Gerais, Rua Dois, Campus Morro do Cruzeiro, Ouro Preto, Ouro Preto, MG CEP 35.400-000 Brasil; 2 Universidade Federal do Triângulo Mineiro. Programa de Pós Graduação em Atenção à Saúde, Av. Frei Paulino, nº 30 - Bairro Abadia, Uberaba, Minas Gerais CEP: 38025-180 Brasil; 3 Escola de Educação Física e Desportos, Universidade Federal do Rio de Janeiro, Av. Carlos Chagas Filho, 540 - Cidade Universitária da Universidade Federal do Rio de Janeiro, Rio de Janeiro - RJ, Rio de Janeiro, RJ CEP: 21941-599 Brasil; 4 Universidade Federal do Triângulo Mineiro, Programa de Pós Graduação em Atenção à Saúde, Av. Frei Paulino, nº 30 - Bairro Abadia, Uberaba, Minas Gerais CEP: 38025-180 Brasil; 5 Instituto Nacional de Infectologia Evandro Chagas (INI/FIOCRUZ), Av. Brasil, 4036, sala 201 A - Manguinhos, Rio de Janeiro, CEP: 21040-360 Brasil; 6 Escola Nacional de Saúde Pública Sergio Arouca (ENSP/FIOCRUZ), Rua Leopoldo Bulhões, 1480 - Manguinhos, Rio de Janeiro, Brasil

**Keywords:** Obesity, COVID-19, Pandemics

## Abstract

Previous studies have shown that associations between obesity and other comorbidities favor worse outcomes in COVID-19. However, it is not clear how these factors interrelate and whether effects on men and women differ. We conducted an observational, cross-sectional study using a national COVID-19 inpatient database. We studied differences in direct and indirect effects of obesity and comorbidities according to sex and body mass index (BMI) categories in hospitalized COVID-19 patients in Brazil using path analysis models and logistic regression. For men, path analysis showed a direct association between BMI and death and a negative correlation of death and chronic cardiovascular disease (CCD). For women, the association of BMI and death was indirect, mediated by admission to the ICU and comorbidities and association with CCD was non-significant. In the logistic regression analyses, there was a positive association between death and BMI, age, diabetes mellitus, kidney and lung diseases and ICU admission. We highlight the need to consider the distinct impact of obesity and sex on COVID-19, of monitoring of BMI and of the design for specific male-targeted approaches to manage obesity.

**Supplementary Information:**

The online version contains supplementary material available at 10.1186/s12889-023-16218-1.

## Introduction

By June 2021, 6,493,773 COVID-19 deaths and 603,927,234 infected people had been reported worldwide [1]. By then, Brazil had 34,456,145 confirmed cases and 684,262 deaths, with 2.0% mortality rate [2] turning the country into one of the worst hit by the pandemic.

The severity of COVID-19 symptoms varies widely, ranging from mild or even asymptomatic to very severe, leading to death [3]. Among the main risk factors associated with the poor prognosis of COVID-19, advanced age, male sex and the presence of comorbidities stand out, with emphasis on cardiovascular diseases, lung diseases, diabetes, kidney disease, chronic liver disease and obesity [4, 5]. Body Mass Index (BMI) above 30 kg/m^2^ is associated with unfavorable outcomes in patients infected with the new coronavirus [5–7].

In Brazil, 34.4% of the adult population is overweight and 25.9% has obesity [8]. Obesity is related to changes in breathing dynamics favoring complications in COVID-19, such as increased demand for ventilation and breathing effort. These changes increases chances of more severe breathing infections [6, 9]. Obesity was also found to be a strong independent factor for respiratory failure, UCI admission and death among COVID-19 patients [10].

The physiopathology of obesity involves an inflammatory state associated with chronic activation of the immune system, which negatively affects immune functions. This engenders a large release of cytokines and chemokines and the recruitment of cells that produce pro-inflammatory molecules, with increased immune responses and high rates of multisystemic complications [11]. The expression of the angiotensin-converting enzyme (ACE2) gene also seems to be increased in the subcutaneous and visceral adipose tissue, favoring the entry of the COVID-19 virus into the host cell and worsening the infection in patients with obesity [12].

Besides acting as an independent factor for unfavorable outcomes in COVID-19 infection, obesity is a risk factor for cardiovascular diseases, diabetes, high blood pressure and different kinds of cancer, which are in themselves risk factors for worse outcomes in this disease [13]. This raises initial issues on the downstream associations between obesity and obesity-related comorbidities, which can also independently impact on the course of the COVID-19 infection.

An additional question concerns differences between sexes. Even though data from the *Global Health 50/50 research initiative* identifies similar incidences (cases) of COVID-19 in men and women, men have more unfavorable outcomes and higher mortality rates in SARS-CoV-2 infection [14]. Several studies carried out in China and European countries show males are at higher risk for more severe disease outcomes and mortality [15–17]. This difference in severity and mortality between sexes has been hypothesized to be due to the higher prevalence of specific comorbidities in men, especially hypertension, cardiovascular disease (CVD), and chronic obstructive pulmonary disease (COPD) [17, 18]. Some studies highlight that male patients seem to be more vulnerable to the presence of comorbidities in COVID-19 compared to female patients [19].

A specific mention to the relevance of gender, as opposed to sex as a biological variable, should be made here. Literature has highlighted for some time the importance of considering both the biological-oriented perspective of sex and the more psychosocially centered perspective of gender in clinical studies [20]. However, there are substantial challenges involved in obtaining gender-related information in quantitative studies using administrative databases. So, in this study we are assuming differences considering sex, as opposed to gender.

It is still unclear how obesity and sex function as direct risk factors or through the mediation of comorbidities favoring worse outcomes in COVID-19. A better understanding of differences in outcomes between sexes may support the development of risk classification tools and of treatment and prevention options for men and women [21].

Structural equation modeling (SEM) [22] allows for the simultaneous analysis of the dependence and interrelationships between multiple variables. It estimates the direct effects and effects mediated by other factors that make up the causal network of the outcome of interest [22]. Path analysis is a special application of SEM [23] requiring large N-datasets. National health surveillance data samples obtained from countries with high incidence of COVID-19 and well-structured administrative information systems, such as Brazil, provide an opportunity for initial explorations of links or pathways between explanatory variables and outcomes using this approach.

The purpose of this study is, based on data recorded in Brazil, to assess mortality from COVID-19 in people with obesity and to describe direct and indirect effects of age, BMI, and clinical variables, according to sex.

## Methods

This observational, cross-sectional study was carried out using microdata from the Influenza Epidemiological Surveillance Information System, SIVEP-Gripe (*Sistema de Informação de Vigilância Epidemiológica da Gripe*- https://sivepgripe.saude.gov.br/sivepgripe/login.html?0). The system is managed by the Brazilian Ministry of Health, in partnership with the State and Municipal Health Secretariats and is used to monitor Severe Acute Respiratory Syndrome (SARS) of any cause (influenza, COVID-19, other respiratory viruses and other or non-specified agents) [24]. Reporting of all cases is mandatory for every public and private healthcare provider in the country.

The variables were selected from the individual record form - Case of Síndrome Respiratória Aguda Grave (inpatient SRAG) at SIVEP-Gripe [25]. The final study database included all confirmed cases of COVID- related severe acute respiratory syndrome reported between 02/21/2020 and 04/18/2021 in the SIVEP- Gripe original dataset featuring obese individuals aged 20 years and over, with recorded BMIs values for obesity (BMI ≥ 30 kg/m^2^). Confirmation and reporting of cases followed criteria of the Epidemiological Surveillance Guide of the Healthy Ministry for COVID-19: clinical, clinical-epidemiological, clinical-imaging and laboratory criteria [25]. The final dataset used in the study will be provided upon reasonable request to the authors.

As we were intending to compare differences between common variables according to sex, we excluded pregnant and postpartum women. To allow us to maintain a large N-data set, we dropped variables initially considered for the model with substantial missing information (e.g., years of school attendance as a social determinant). The goal of model specification is to identify a testable and useful model, even if it fails to account for all aspects of reality [23, 26].

We performed an initial description of the variables using frequencies, percentages, odds ratio and 95% confidence intervals stratified according to sex (male/female). Variables considered included age, race/color (regrouped as white/non-white), presence or absence of risk factors (diabetes mellitus, chronic lung disease, asthma, chronic kidney disease and chronic cardiovascular disease), presence of obesity, BMI (kg/m^2^), admission to the ICU (yes/no), time from initial symptoms to final outcome (in days) based on dates informed in the database and clinical outcome (cure/death) [25]. There are no specific instructions for reporting the variable sex in SIVEP. It is probably based on assignment by the health care professional filling in the SIVEP forms and not on self-reporting [27].

To assess the direct and indirect associations of mortality from COVID-19 in people with obesity according to sex, we developed a hypothetical model, considering observed variables with valid frequencies above 5% and significance.

The model tested through trajectory analysis (*Path Analysis*) [28] included the variables age, comorbidities (chronic cardiovascular disease, diabetes, chronic renal disease and chronic respiratory disease) and admission to the ICU. Observed variables are represented by rectangles and classified as exogenous and endogenous. The endogenous variables were assigned measurement errors identified by “e”. Specifically, for this analysis, we considered BMI as a continuous variable. We estimated the parameters using asymptomatic distribution free method. The results were presented using direct and indirect standardized regression coefficients. We obtained the indirect standardized coefficients by multiplying the coefficients of the direct tracks among the variables. The goodness of fit of the models was evaluated according to the Chi-square test (χ2) p > 0.05; Root Mean Error of Approximation (RMSEA) ≤ 0.05; Goodness of Fit Index (GFI), Comparative Fit Index (CFI) and Tucker-Lewis Index (TLI) ≥ 0.95 [22].

To characterize the participants and analyze the data, BMI was categorized as: 30.0 to 34.9; 35.0 to 39.9; 40.0 to 49.9 and ≥ 50.0 kg/m^2^ [29]. To determine the association of BMI with mortality according to sex, we used binary logistic regression adjusted for the variables age, comorbidities, and admission to the ICU, with estimates of *odds ratios* (ORs) with the corresponding 95% confidence intervals (CI).

Direct standardized coefficients and p-values were determined for all variables included in the model. A path coefficient indicates the direct effect of a variable assumed to be the cause on another variable, assumed to be the effect. Standardized coefficients allow for the comparison among the relative importance of different variables assessed by adjusting the standard deviations in such a way that, despite different units of measurement, all the variables have equal standard deviations. They are sample specific and cannot be compared across samples of studies.

For logistic regression analyses, where coefficients allow comparisons across different data samples, we determined odds Ratio (OR) and confidence interval (95%CI) for the association of age and clinical variables with mortality from COVID-19 among people with obesity, according to sex.

We conducted all analyzes using IBM SPSS and IBM SPSS AMOS 24.

In accordance with Resolution 466/2012 of the Brazilian National Health Council, approval of the study by the Research Ethics Committee was not required, as it uses public open access unidentified data.

## Results

During the study period, 1,048,576 individuals were reported in the SIVEP-Gripe inpatient database. Among the 50,197 reported as obese, BMIs in the expected range of 30- 99.9 kg/m^2^ were available for 13,494 (26.9%). Our study dataset, considering the exclusion criteria, included 8,772 adults with a diagnosis of COVID-19- related SRAG and an available BMI value indicating obesity (BMI > 30).

Table 1 shows the descriptive analysis of the variables according to sex. In our study population, 51.7% were men and 52.1% were white. Average age was 54.4 years old (SD = 15.02) and average BMI 35.5 (SD = 6.09) kg/m^2^. Reported deaths amounted to 3,115 (35.5% of patients), with similar mortality for males (35.7%) and females (35.4%). Most patients had BMI in the range of 30.0 to 34.9 kg/m^2^ (n = 4,420; 50.4%) and were admitted to the ICU (n = 4,447; 50.7%). Regarding comorbidities, 49.7% had chronic cardiovascular disease, 37.4% diabetes mellitus, 6.7% asthma, 5.6% chronic lung disease, and 5.4% chronic kidney disease. Times from symptoms to outcomes did not differ between sexes (Table 1).

Overall frequencies for other less frequent comorbidities were neurologic disease (3.2%); blood disease (1%); Down´s syndrome (0.6%); liver disease (1.4%); and immune deficiency (2.7%) (not shown).

**Table 1:** Characteristics of individuals with obesity with confirmed COVID-19-related severe acute respiratory syndrome, according to sex, Brazil, 2020-1

Variables	Men (n = 4,535; 51.7%)	Women (n = 4,237; 48.3%)
Age in years, average, (SD)	52.3 (14.2)	56.7 (15.6)
BMI in kg/m^2^, average (SD)	35.4 (5.9)	35.6 (6.3)
**Color/race**		
White	2,340 (51.6)	2,228 (57.6)
Non-white	2,195 (48.4)	2,008 (47.4)
**BMI, n (%) (kg/m** ^**2**^ **)**		
30,0–34,9	2,316 (51.1)	2,104 (49.7)
35,0–39,9	1,296 (28.6)	1,208 (28.5)
40,0–49,9	810 (17.9)	800 (18.9)
≥ 50,0	113 (2.5)	125 (3.0)
**Comorbidities n (%)**		
Chronic Cardiovascular Disease	2,102 (46.4)	2,259 (53.3)
Diabetes *mellitus*	1,540 (34.0)	1,737 (41.0)
Chronic Kidney Disease	212 (4.7)	277 (6.5)
Asthma	218 (5.6)	371(10.8)
**Clinical course**		
Symptoms to outcome (days/SD)	20.4 (15.2)	21.1(16.4)
ICU (%) **Outcome (%)**	2,413 (53.2)	2,034 (48.0)
Death	1,617 (35.7)	1,498 (35.4)

BMI: Body Mass Index; SD: Standard deviation; ICU: Intensive Care Unit

**Fig. 1:**
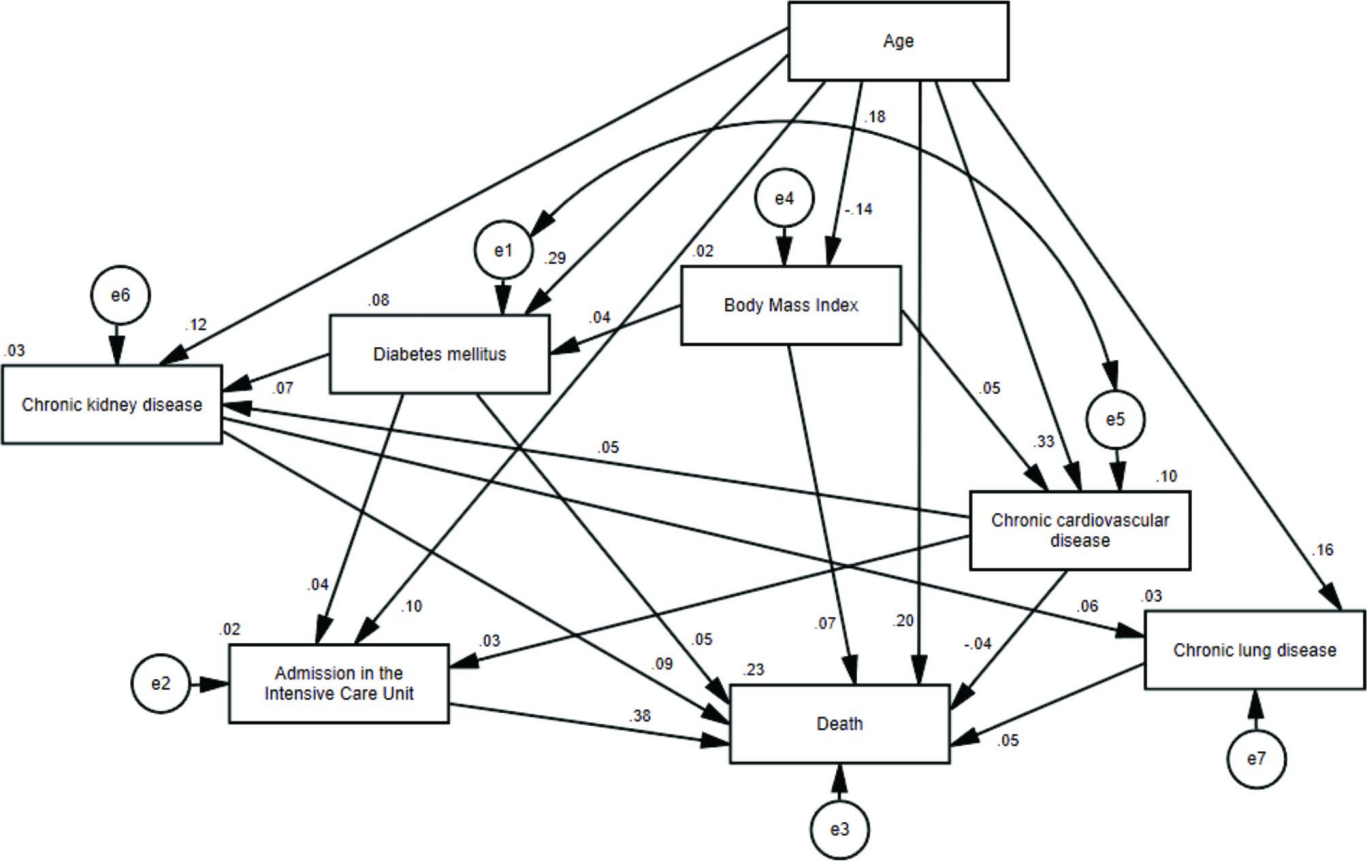
Model of the association between body mass index and death for male patients with obesity diagnosed with Covid-19 (χ2 = 8.245; p = 0.311; RMSEA = 0.006; CFI = 0.999; GFI = 1.000; TLI = 0.998)

**Fig. 2:**
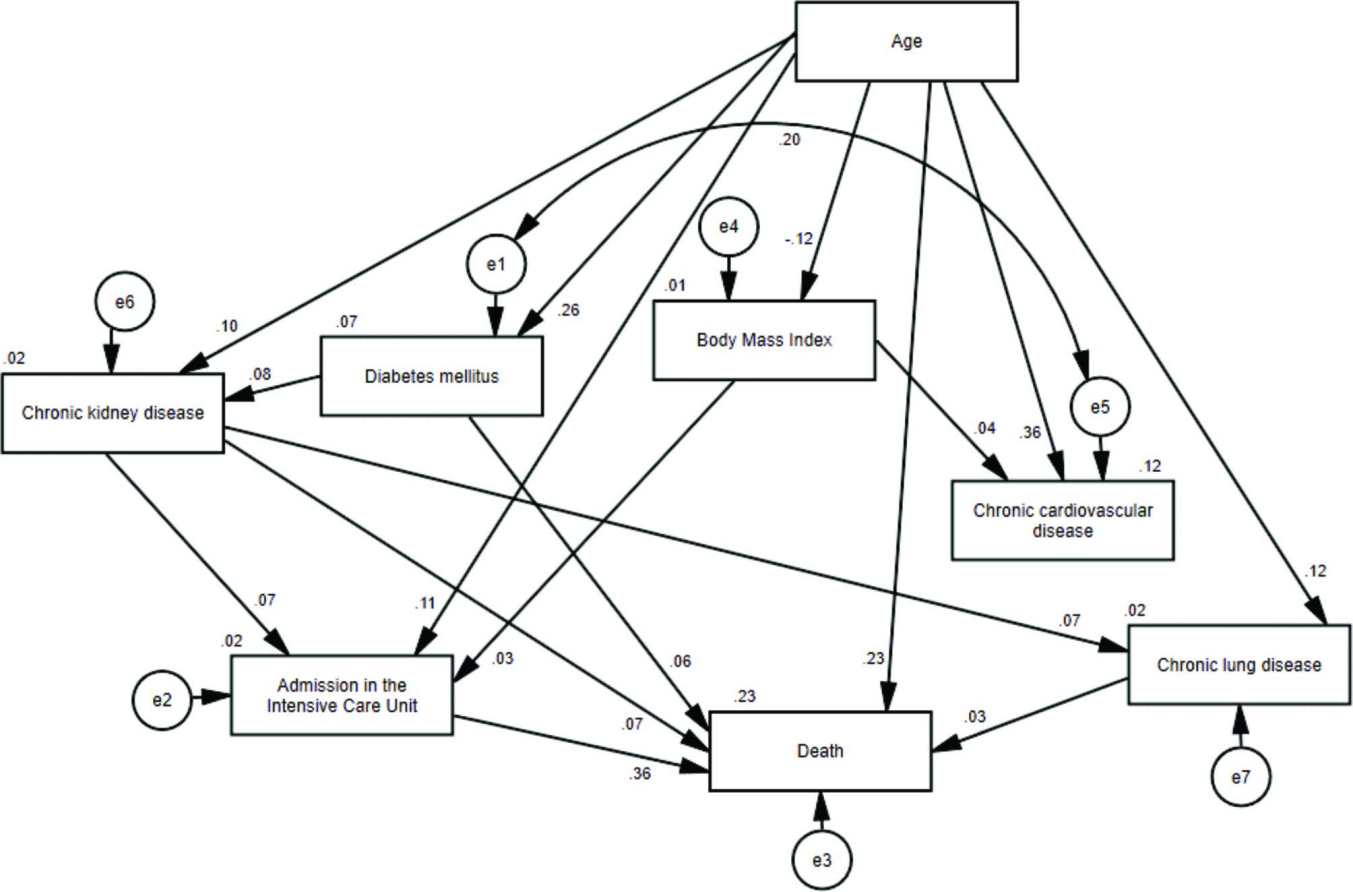
Model of the association between body mass index and death for female patients with obesity diagnosed with Covid-19 (χ2 = 8.245; p = 0.311; RMSEA = 0.006; CFI = 0.999; GFI = 1.000; TLI = 0.998)

We performed path analyzes using models for male and female patients. Both models presented acceptable goodness-of-fit indices (Figs. 1 and 2).

Using standardized path coefficients and considering the outcome death (Table 2), the greatest direct effects in both men and women were related to admission to ICU (0.38 for men and 0.36 for women) and age (0.20 for men and 0.23 for women). BMI showed a significant correlation to death in men (0.07) but not in women. Also, chronic cardiovascular disease was only significant in men, but shows a negative correlation (-0.04). Since none of the coefficients exceeded one, we could conclude that there was no multicollinearity.

**Table 2:** Direct standardized coefficients for variables* analyzed in the model of the association between body mass index and death, according to sex, Brazil, 2020-1

Direct effects	Estimate	p-value
**Men**		
**Age to:**		
*Chronic Kidney Disease*	0.12	< 0.001
*Diabetes mellitus*	0.29	< 0.001
*Body Mass Index*	-0.14	< 0.001
*Chronic Cardiovascular Disease*	0.33	< 0.001
*Chronic Lung Disease*	0.16	< 0.001
*Admission to the Intensive Care Unit*	0.10	< 0.001
*Death*	0.20	< 0.001
**Chronic Kidney Disease to**:		
*Chronic Lung Disease*	0.06	0.008
*Death*	0.09	< 0.001
**Diabetes mellitus to**:		
*Chronic Kidney Disease*	0.07	< 0.001
*Admission to the Intensive Care Unit*	0.04	0.012
*Death*	0.05	< 0.001
**Body Mass Index to**:		
*Diabetes mellitus*	0.04	0.002
*Chronic Cardiovascular Disease*	0.05	0.003
*Death*	0.07	< 0.001
**Chronic Cardiovascular Disease to**:		
*Chronic Kidney Disease*	0.05	0.002
*Admission to the Intensive Care Unit*	0.03	0.030
*Death*	-0.04	0.005
**Admission to the Intensive Care Unit to**:		
*Death*	0.38	< 0.001
**Chronic Lung Disease to**:		
*Death*	0.05	< 0.001
**Women**		
**Age to**:		
*Chronic Kidney Disease*	0.10	< 0.001
*Diabetes mellitus*	0.24	< 0.001
*Body Mass Index*	-0.12	< 0.001
*Chronic Cardiovascular Disease*	0.36	< 0.001
*Chronic Lung Disease*	0.12	< 0.001
*Admission to the Intensive Care Unit*	0.11	< 0.001
*Death*	0.23	< 0.001
**Chronic Kidney Disease to**:		
*Admission to the Intensive Care Unit*	0.07	< 0.001
*Chronic Lung Disease*	0.07	< 0.001
*Death*	0.07	< 0.001
**Diabetes mellitus to**:		
*Chronic Kidney Disease*	0.08	< 0.001
*Death*	0.06	< 0.001
**Body Mass Index to**:		
*Admission to the Intensive Care Unit*	0.03	< 0.001
*Chronic Cardiovascular Disease*	0.04	0.018
**Chronic Lung Disease to**:		
*Death*	0.03	0.028
**Admission to the Intensive Care Unit to**:		
*Death*	0.36	< 0.001

*Only significant relationships between variables are shown

In the model tested in men with obesity, older age (β = 0.20), higher BMI values (β = 0.07), admission to the ICU (β = 0.38), presence of diabetes mellitus (β = 0.05), chronic kidney disease (β = 0.09), chronic lung disease (β = 0.05) and chronic cardiovascular disease (β= -0.04) were associated with death.

For women with obesity, we identified a direct association between older age (β = 0.23). admission to the ICU (β = 0.36), presence of diabetes mellitus (β = 0.06), chronic kidney disease (β = 0.07), chronic lung disease (β = 0.03) and death. BMI was indirectly associated and was mediated by admission to the ICU (β = 0.012).

Logistic regression analysis after adjustment showed that age, diabetes, chronic kidney disease, chronic lung disease and admission to ICU were associated with death in patients of both sexes (Table 3). Chronic cardiovascular disease was only associated with death in male patients, with an OR of 0.81 (95%CI = 0.70–0.94). After adjustment for age, chronic cardiovascular disease, diabetes mellitus, chronic lung disease, chronic kidney disease and admission to ICU, elevated BMI was associated with death in both men and women (Table 2). Male patients with a BMI between 40 and 49.9 kg/m^2^ and ≥ 50.0 kg/m^2^ had greater chance of death when compared with those with a BMI between 30 kg/m^2^ and 34.9 kg/m^2^, with ORs of 1.50 (95%CI = 1.32–1.94) and 3.0 (95%CI = 1.94–4.64), respectively. In women, an association was found only for BMI ≥ 50.0 kg/m^2^, with 1.81 (95%CI = 1.19–2.77) more chance of death compared to those with a BMI between 30 kg/m^2^ and 34.9 kg/m^2^.

**Table 3:** Odds Ratio (OR) and confidence interval (95%CI) for the association of age and clinical variables with mortality from COVID-19 among people with obesity, according to sex. Brazil. 2020-1

Variables	Death			
	Men OR (IC95%)*	p-value	Women OR (IC95%)*	p-value
**Age in Years**	1.04 (1.03–1.04)	< 0.001	1.04 (1.03–1.05)	< 0.001
**Color**				
White	1		1	
Non White	1.48 (1.26–1.74)	< 0.001	1.66 (1.41–1.96)	< 0.001
**Symptoms to outcome (days)**	0.98 (0.98–0.99)	0.79	0.98 (0.98–0.99)	< 0.001
**Chronic Cardiovascular Disease**		0.01		0.07
No	1		1	
Yes	0.81 (0.69–0.96)		1.17 (0.99–1.39)	
**Diabetes** ***mellitus***		< 0.001		< 0.001
No	1		1	
Yes	1.32 (1.15–1.55)		1.29 (1.15–1.52)	
**Chronic Kidney Disease**		< 0.001		< 0.001
No	1		1	
Yes	2.32 (1.64–3.29)		1.75 (1.25–2.45)	
**Chronic Lung Disease**		0.001		0.021
No	1		1	
Yes	1.87 (1.29–2.69)		1.49 (1.10–2.04)	
**Asthma**		0.88		0.84
No	1		1	
Yes	1.22 (0.84–1.76)		1.05 (0.79–1.40)	
**Admission to ICU**		< 0.001		< 0.001
No	1		1	
Yes	7.40 (6.25–8.88)		6.88 (5.79–8.12)	
**BMI (kg/m** ^**2**^ **)**		< 0.001		0.017
30.0 a 34.9	1		1	
35.0 a 39.9	1.10 (0.92–1.31)		0.92 (0.77–1.39)	
40.0 a 49.9	1.50 (1.22–1.85)		1.03 (0.82–1.27)	
≥ 50.0	3.00 (1.91–4.94)		1.69 (1.06–2.71)	

ICU: Intensive Care Unit; BMI: Body Mass Index. *Adjusted by all variables included in regression models.

In the logistic regression analysis, where coefficients allow comparisons across different data samples, differences between sexes in increased chances of dying were very similar in diabetes mellitus (32% for men and 29% for women) but differed for chronic lung disease (men 87% and women 49%) and chronic kidney disease (132% for men and 75% for women).

Worsening of the disease and admission to the ICU remained the biggest risk factor for mortality for people with obesity. Upon admission to ICU, the chance of dying increased 7.4 times in men and 5.88 times in women when compared to no admission to the ICU.

## Discussion

Initial descriptions of frequencies for COVID-19 mortality, disease duration and UCI use in our study did not hint at substantial differences between sexes. However, pathway analysis shows that obesity in men has a direct effect on COVID-19 deaths, not mediated by comorbidities. Obesity is, thus, a key factor in unfavorable COVID outcomes in men. On the other hand, we did not find this direct relationship of BMI and death in women. In their case, death was indirectly related to co-morbidities and by the need for admission to the ICU. Comorbidities and admission to ICU seemed, thus, to be more “decisive” than obesity for the outcome death in COVID-19 infected women.

As shown in the logistic regression analyses, for women with BMIs in the extreme obesity range (> 50 Kg/m^2^) obesity increased the risk of COVID-19 death by 69%. Men in the same BMI range showed a three times higher magnitude of association (200%). Men also evidenced an increased risk for COVID-19 mortality at lower levels of obesity (> 40 kg/m^2^) compared to women. As the reference for comparison in our study was the 30–35 kg/m^2^ BMI range, the actual magnitude of the effect tends to be even greater. Previous studies using < 30 kg/m^2^ BMI as reference showed increases in mortality of 27% relative to mild obesity (30–35 kg/m^2^ BMI) [30].

Obesity alone is a more significant risk factor than cardiovascular disease and diabetes for in-hospital COVID-19 mortality [31] and should be treated as a crucial biological factor [32]. This creates a very worrisome context in countries with high prevalence of obesity and where the pandemic remains out of control for long spells. Approximately 34 million Brazilians ≥ 50 years of age have at least one risk condition for severe COVID-19, obesity being among three most prevalent [33]. Current evidence already suggests the need to prioritize obese young individuals for vaccination should COVID-19 persist as a recurrent seasonal infection [31]. Our study suggests the need for special emphasis on men.

Male sex was also found to be an independent risk factor for COVID-19 disease in previous studies using Brazilian data [32]. The greater vulnerability of men with obesity to death from coronaviruses independently of other comorbidities calls for strengthening gender-oriented public-health recommendations concerning the risks of obesity [34]. In many countries, traditional gender perspectives on aesthetics undervalue and even discourage concerns over body weight in men. The quest for beauty, heavily linked to slimness, addresses mainly women. Public health strategies for obesity in men may, thus, pose greater challenges than in the case of women.

The availability of consistent BMI information for little over one quarter of patients reported as obese in SIVEP-Gripe signals the need to emphasize the importance of this measurement. This would include training healh care professionals on how to measure BMI or on how to get quick information from online apps. As previously shown in a study on hypertension, in addition to minding gender differences in risk awareness, emphasis on measurement tends to favour a more adequate monitoring of risk factors [35].

An unexpected finding in our study was the counterintuitive result for risk of death from COVID-19 found for obese men reporting chronic cardiovascular disease (CCD). A recent study came to suggest a possible key role of cardiovascular disease in COVID-19 by showing that the age pattern of the male-to-female ratio in mortality closely mirrors that of overall mortality from cardiovascular disease [36]. Several other studies also cite CCD as a risk factor in both men and women [37].

Previous Brazilian studies using the SIVEP-Gripe have nevertheless detected non-significant, negative, or dubious impacts of CCD on COVID-19 deaths. Souza et al. (2020). used an adjusted Cox regression model to stablish associations of CCD with fatal outcomes in COVID-19 and found a HR of 0.95 (0.93–0.98) [38, 39]. Hallak et al. (2022), using microdata for São Paulo found negative and statistically significant coefficients for heart disease in logistic regression models for death from COVID-19 [32]. Another study in a São Paulo hospital analyzed a very stratified set of cardiovascular conditions in 2,675 patients to better understand associations between CCD and COVID-19 deaths (including hypertension, arrhythmias, coronary artery disease, previous myocardial infarction, cardiac surgery and valve and congenital heart diseases). It confirmed that none of them acted as independent risk factors for death [40].

Outside Brazil, a survey using a large UK National Health Service (NHS) dataset also showed that neither diagnosed hypertension nor high blood pressure impacted patient´s outcomes in COVID. with marginal or small effects for other preexisting CCD [41]. All these findings suggest that the relevance of cardiovascular disease in unfavorable COVID-19 outcomes may occur in the context of acute cardiovascular events developed in the course of the disease and not necessarily of preexisting chronic heart conditions [40]. However, the contradictions between results in studies examining the relevance of cardiovascular disease in COVID-19 severity emphasize the need to revisit these relationships in future research to better understand the trends.

Hypertension, atherosclerosis-related and other causes of chronic cardiovascular disease are grouped under the single variable CCD in the SIVEP-Gripe. Several studies have shown possible differences in the contributions of hypertension [42] and other cardiovascular diseases to COVID-19 severity [43]. Given the large N-data set in SIVEP-Gripe and its evident usefulness in clinical and epidemiological studies, being able to distinguish arterial hypertension from other CCDs would contribute to better illuminate the relationships between CCD and death in COVID-19 [43]. The Mexican Epidemiologic Surveillance Database for Respiratory Viral diseases (Sistema de Vigilancia Epidemiológica de Enfermedades Respiratorias Virales), for instance, distinguishes hypertension from cardiopathy. In a study using this specific database hypertension was associated with increased risk of death from COVID-19, whereas cardiopathy was not [44].

Literature confirms our regression analysis findings that age [45, 46], diabetes mellitus [47–49], chronic kidney disease [50, 51], chronic lung disease [4, 52, 53] and admission to ICU [50, 51] are all associated with COVID-19 mortality in people with obesity of both sexes. For chronic lung disease the effect was modest but statistically significant, with slight difference between sexes (odds ratio varying from 1.5 for men to 1.6 in women). The greatest risk for adverse health outcomes was admission to the ICU [54–56]. Chronic kidney disease (CKD) was the second most important independent risk factor for COVID-19 mortality. It increased women´s risk by 39% and by 149% in men. This result supports the adoption in Brazil of special measures for COVID-19 protection in dialysis environments during outbreaks [56], wider use of screening to detect early-stage kidney disease and campaigns to inform the public and health professionals of increased vulnerability to severe Covid-19 and other infections in CKD [57].

Differences in male vulnerability to COVID-19 [32] and the many hypotheses concerning distinct demands, behaviors and ways of life incident upon men highlight the importance of considering the gender approach in future studies [20, 58]. On the other hand, links between testosterone, obesity and responses to COVID-19 emphasize biological aspects of the differences between sexes. The relationship between testosterone and COVID-19 seems to be dependent upon the specific disease stage, but there is a well-stablished association between low levels of testosterone, SARS-COV2-induced oxidative stress and greater disease severity [59]. The obesity-associated hypotestosteronemia seen in moderate and severe male obesity [60] may thus predispose to worse outcomes in COVID-19.

Obesity-associated hypotestosteronemia by itself leads to a vicious cycle of increasing adiposity. It remains a functional and reversible status but requires substantial weight loss to be reversed [60]. In moderate and severe obesity this may only be achievable by bariatric surgery or modern anti-obesity drugs. Therefore, one cannot overstate the importance of starting to manage overweight and mild obesity in primary care settings. Since 2009 there is a national program for men´s health improvement delivered at primary health care facilities in Brazil [61]. The inclusion of obesity control targets [62] in this program could contribute to developing gender-sensitive approaches to obesity.

This study has limitations and strengths. The considerable number of missing data stands out as a limitation. Since filling instructions in administrative records are often not sufficiently precise by academic research standards, there are no guarantees regarding the control of filling in the data and associated biases. Despite the adequate fit, our model is not unique and there may be other valid ways to explain the relationships under study. Additionally, since the interaction of COVID-19 with other variables is still under study, erroneous causal relationships may have been established in the process of designing the hypothetical model. As strengths, we highlight the use of the large-N free access national database of hospitalized patients with COVID-19 and the specific perspective of comparison between sexes adopted in our study.

In conclusion, as short-term approaches in COVID outbreaks, our study supports strict protective measures to avoid infection and priority vaccination in men with BMI > 40 kg/m^2^, especially in those with CKD and chronic lung disease. Longer-term public health recommendations would include systematic monitoring of BMI and fostering adequate gender sensitive approaches to the management of obesity in primary care.

### Electronic supplementary material

Below is the link to the electronic supplementary material.

Supplementary Material 1

## Data Availability

The datasets analysed during the current study are available in the Banco de Dados de Síndrome Respiratória Aguda Grave - incluindo dados da COVID-19. https://opendatasus.saude.gov.br/dataset/srag-2021-e-2022.

## References

[1] (2021) WHO Coronavirus (COVID-19) Dashboard. https://covid19.who.int. Accessed 25 May 2021.

[2] (2021) Coronavírus Brasil. https://covid.saude.gov.br/. Accessed 24 May 2021.

[3] Weiss P, Murdoch DR (2020). Clinical course and mortality risk of severe COVID-19. Lancet Lond Engl.

[4] CDC. (2020) COVID-19 and Your Health. In: Cent. Dis. Control Prev. https://www.cdc.gov/coronavirus/2019-ncov/need-extra-precautions/people-with-medical-conditions.html. Accessed 25 May 2021.

[5] de Siqueira JVV, Almeida LG, Zica BO, Brum IB, Barceló A, de Siqueira Galil AG (2020). Impact of obesity on hospitalizations and mortality, due to COVID-19: a systematic review. Obes Res Clin Pract.

[6] Michalakis K, Panagiotou G, Ilias I, Pazaitou-Panayiotou K (2021). Obesity and COVID-19: a jigsaw puzzle with still missing pieces. Clin Obes.

[7] Zhou Y, Yang Q, Chi J, Dong B, Lv W, Shen L, Wang Y (2020). Comorbidities and the risk of severe or fatal outcomes associated with coronavirus disease 2019: a systematic review and meta-analysis. Int J Infect Dis IJID Off Publ Int Soc Infect Dis.

[8] IBGE (2020). Pesquisa Nacional de Saúde: 2019: Atenção primária à saúde e informações antropométricas: Brasil.

[9] Parameswaran K, Todd DC, Soth M (2006). Altered respiratory physiology in obesity. Can Respir J.

[10] Rottoli M, Bernante P, Belvedere A (2020). How important is obesity as a risk factor for respiratory failure, intensive care admission and death in hospitalised COVID-19 patients? Results from a single italian centre. Eur J Endocrinol.

[11] Zhou Y, Chi J, Lv W, Wang Y (2021). Obesity and diabetes as high-risk factors for severe coronavirus disease 2019 (Covid-19). Diabetes Metab Res Rev.

[12] Al-Benna S (2020). Association of high level gene expression of ACE2 in adipose tissue with mortality of COVID-19 infection in obese patients. Obes Med.

[13] World Health Organization. (2000) Obesity: preventing and managing the global epidemic: report of a WHO consultation. 252–252. 11234459

[14] The Sex., Gender and COVID-19 Project | Global Health 50/50.

[15] Gebhard C, Regitz-Zagrosek V, Neuhauser HK, Morgan R, Klein SL (2020). Impact of sex and gender on COVID-19 outcomes in Europe. Biol Sex Differ.

[16] Meng Y, Wu P, Lu W (2020). Sex-specific clinical characteristics and prognosis of coronavirus disease-19 infection in Wuhan, China: a retrospective study of 168 severe patients. PLOS Pathog.

[17] Qin L, Li X, Shi J (2020). Gendered effects on inflammation reaction and outcome of COVID-19 patients in Wuhan. J Med Virol.

[18] Grasselli G, Zangrillo A, Zanella A (2020). Baseline characteristics and outcomes of 1591 patients infected with SARS-CoV-2 admitted to ICUs of the Lombardy Region, Italy. JAMA.

[19] Jin J-M, Bai P, He W, Wu F, Liu X-F, Han D-M, Liu S, Yang J-K (2020). Gender differences in patients with COVID-19: focus on severity and mortality. Front Public Health.

[20] Clayton JA, Tannenbaum C (2016). Reporting sex, gender, or both in Clinical Research?. JAMA.

[21] Klein SL, Dhakal S, Ursin RL, Deshpande S, Sandberg K, Mauvais-Jarvis F (2020). Biological sex impacts COVID-19 outcomes. PLOS Pathog.

[22] Marôco J (2014). Análise de Equações Estruturais: Fundamentos teóricos, software & Aplicações.

[23] Hoyle RH. (2012) Path analysis and structural equation modeling with latent variables. In: Cooper H, Camic PM, Long DL, Panter AT, Rindskopf D, Sher KJ, editors APA Handb. Res. Methods Psychol. Vol 2 Res. Des. Quant. Qual. Neuropsychol. Biol. American Psychological Association, Washington, pp 333–367.

[24] Brasil, Secretaria de Vigilância em Saúde. (2021) Ministério da Saúde. Secretaria de Vigilância em Saúde. Guia de vigilância epidemiológica: emergência de saúde pública de importância nacional pela doença pelo coronavírus 2019 – covid-19.

[25] (2021) SRAG 2020 - Banco de Dados de Síndrome Respiratória Aguda Grave - incluindo dados da COVID-19 - Open Data. https://opendatasus.saude.gov.br/dataset/bd-srag-2020. Accessed 25 May 2021.

[26] Heidari S, Babor TF, De Castro P, Tort S, Curno M (2016). Sex and gender equity in Research: rationale for the SAGER guidelines and recommended use. Res Integr Peer Rev.

[27] Brasil, Secretaria de Vigilancia em Saúde, SIVEP Gripe - SISTEMA DE INFORMAÇÃO DE VIGILÂNCIA EPIDEMIOLÓGICA DA GRIPE FICHA DE REGISTRO INDIVIDUAL. (2021) Ficha de Notificação da Vigilância da Síndrome Respiratória Aguda Grave (SRAG) no Brasil. - CASOS DE SÍNDROME RESPIRATÓRIA AGUDA GRAVE HOSPITALIZADO- 27/07/2020.

[28] Kline RB. (2011) Principles and practice of structural equation modeling, 3rd ed. xvi, 427.

[29] Standards Committee AS for BS (1997). Guidelines for reporting results in bariatric surgery. Obes Surg.

[30] Palaiodimos L, Kokkinidis DG, Li W, Karamanis D, Ognibene J, Arora S, Southern WN, Mantzoros CS (2020). Severe obesity, increasing age and male sex are independently associated with worse in-hospital outcomes, and higher in-hospital mortality, in a cohort of patients with COVID-19 in the Bronx, New York. Metabolism.

[31] Discacciati MG, Siani S, Campa A, Nakaya HI (2022). Why should obese youth be prioritized in COVID-19 vaccination programs? A nationwide retrospective study. Lancet Reg Health - Am.

[32] Hallak J, Teixeira TA, Barrozo LV, Singer J, Kallas EG, Saldiva PH (2022). Male sex rather than socioeconomic vulnerability as a determinant for COVID-19 death in Sao Paulo: a population-based study. SAGE Open Med.

[33] Nunes BP, de Souza ASS, Nogueira J, de Andrade FB, Thumé E, Teixeira DSdaC, Lima-Costa MF, Facchini LA, Batista SR (2020). Multimorbidity and population at risk for severe COVID-19 in the *brazilian longitudinal study of aging*. Cad Saúde Pública.

[34] Harris MB, Walters LC, Waschull S (1991). Gender and ethnic differences in obesity-related behaviors and attitudes in a College Sample. J Appl Soc Psychol.

[35] Rahman M, Williams G, Al Mamun A (2017). Gender differences in hypertension awareness, antihypertensive use and blood pressure control in bangladeshi adults: findings from a national cross-sectional survey. J Health Popul Nutr.

[36] Nimgaonkar I, Valeri L, Susser E, Hussain S, Sunderram J, Aviv A (2021). The age pattern of the male-to-female ratio in mortality from COVID-19 mirrors that of cardiovascular disease in the general population. Aging.

[37] Bae S, Kim SR, Kim M-N, Shim WJ, Park S-M (2021). Impact of cardiovascular disease and risk factors on fatal outcomes in patients with COVID-19 according to age: a systematic review and meta-analysis. Heart.

[38] de Souza FSH, Hojo-Souza NS, Batista BD, de O, Silva CM, Guidoni DL (2021). On the analysis of mortality risk factors for hospitalized COVID-19 patients: a data-driven study using the major brazilian database. PLoS ONE.

[39] Kim L, Garg S, O’Halloran A (2021). Risk factors for Intensive Care Unit Admission and In-hospital mortality among hospitalized adults identified through the US Coronavirus Disease 2019 (COVID-19)-Associated hospitalization Surveillance Network (COVID-NET). Clin Infect Dis.

[40] Magalhães BK, Queiroz F, Salomão MLM, de Godoy MF (2022). The impact of chronic cardiovascular disease on COVID-19 clinical course. J Clin Transl Res.

[41] Williamson EJ, Walker AJ, Bhaskaran K (2020). Factors associated with COVID-19-related death using OpenSAFELY. Nature.

[42] Tadic M, Saeed S, Grassi G, Taddei S, Mancia G, Cuspidi C (2021). Hypertension and COVID-19: ongoing controversies. Front Cardiovasc Med.

[43] Haji Aghajani M, Asadpoordezaki Z, Haghighi M, Pourhoseingoli A, Taherpour N, Toloui A, Sistanizad M (2021). Effect of underlying Cardiovascular Disease on the prognosis of COVID-19 patients; a sex and age-dependent analysis. Arch Acad Emerg Med.

[44] Parra-Bracamonte GM, Lopez-Villalobos N, Parra-Bracamonte FE (2020). Clinical characteristics and risk factors for mortality of patients with COVID-19 in a large data set from Mexico. Ann Epidemiol.

[45] Poly TN, Islam MdM, Yang HC, Lin MC, Jian W-S, Hsu M-H, Jack Li Y-C (2021). Obesity and mortality among patients diagnosed with COVID-19: a systematic review and Meta-analysis. Front Med.

[46] Seidu S, Gillies C, Zaccardi F, Kunutsor SK, Hartmann-Boyce J, Yates T, Singh AK, Davies MJ, Khunti K (2020). The impact of obesity on severe disease and mortality in people with SARS-CoV-2: a systematic review and meta-analysis. Endocrinol Diabetes Metab.

[47] CDC COVID-19 Response Team (2020). Preliminary estimates of the prevalence of selected Underlying Health Conditions among patients with Coronavirus Disease 2019 - United States, February 12-March 28, 2020. MMWR Morb Mortal Wkly Rep.

[48] NHS England » Type 1 and Type 2 diabetes. and COVID-19 related mortality in England. https://www.england.nhs.uk/publication/type-1-and-type-2-diabetes-and-covid-19-related-mortality-in-england/. Accessed 6 Jun 2021.

[49] Zhu L, She Z-G, Cheng X (2020). Association of blood glucose control and outcomes in patients with COVID-19 and pre-existing type 2 diabetes. Cell Metab.

[50] Henry BM, Lippi G (2020). Chronic kidney disease is associated with severe coronavirus disease 2019 (COVID-19) infection. Int Urol Nephrol.

[51] Council ERA-EDTA, Working Group (2021). Chronic kidney disease is a key risk factor for severe COVID-19: a call to action by the ERA-EDTA. Nephrol Dial Transplant off Publ Eur Dial Transpl Assoc. Eur Ren Assoc.

[52] Canada PHA. of (2020) Coronavirus disease (COVID-19): Prevention and risks. https://www.canada.ca/en/public-health/services/diseases/2019-novel-coronavirus-infection/prevention-risks.html. Accessed 6 Jun 2021.

[53] Grupos de risco. In: SNS24. https://www.sns24.gov.pt/tema/doencas-infecciosas/covid-19/grupos-de-risco/. Accessed 6 Jun 2021.

[54] Gonçalves DA, Ribeiro V, Gualberto A, Peres F, Luconi M, Gameiro J. (2021) COVID-19 and Obesity: An Epidemiologic Analysis of the Brazilian Data. Int J Endocrinol 2021:e6667135. 10.1155/2021/6667135 PMC8121602 34040642

[55] Kirillov Y, Timofeev S, Avdalyan A, Nikolenko VN, Gridin L, Sinelnikov MY (2021). Analysis of risk factors in COVID-19 adult mortality in Russia. J Prim Care Community Health.

[56] Basile C, Combe C, Pizzarelli F, Covic A, Davenport A, Kanbay M, Kirmizis D, Schneditz D, Van Der Sande F, Mitra S (2020). Recommendations for the prevention, mitigation and containment of the emerging SARS-CoV-2 (COVID-19) pandemic in haemodialysis centres. Nephrol Dial Transplant.

[57] Bridger JC (2022). Raising awareness of acute kidney injury and chronic kidney disease in light of COVID-19. Br J Healthc Manag.

[58] Galbadage T, Peterson BM, Awada J, Buck AS, Ramirez DA, Wilson J, Gunasekera RS (2020). Systematic review and Meta-analysis of sex-specific COVID-19 clinical outcomes. Front Med.

[59] Yassin A, Sabsigh R, Al-Zoubi RM, Aboumarzouk OM, Alwani M, Nettleship J, Kelly D (2023). Testosterone and Covid-19: an update. Rev Med Virol.

[60] Tang Fui M, Dupuis P, Grossmann M (2014). Lowered testosterone in male obesity: mechanisms, morbidity and management. Asian J Androl.

[61] Vasconcelos ICBDL, Prestes JYDN, Ribeiro RRS, Lima SJL, Farias SDCF, Barbosa LDDS, Vasconcelos AC, Duque MAA (2019). Política nacional de atençao integral a saúde do homem e os desafios de sua implementação. Braz J Dev.

[62] Ramos DB das, Burlandy N, Dias L PC, et al. Propostas governamentais brasileiras de ações de prevenção e controle do sobrepeso e obesidade sob perspectiva municipal. Cad Saúde Pública. 2020. 10.1590/0102-311x00116519. 10.1590/0102-311X00116519 32578807

